# Supplementary material for: Fbxo4-mediated degradation of Fxr1 suppresses tumorigenesis in head and neck squamous cell carcinoma
Source: Nat Commun. 2017 Nov 16;8:1534. doi: 10.1038/s41467-017-01199-8 (PMC5688124; doi:10.1038/s41467-017-01199-8)
Supplement: Supplementary file 1 — Supplementary Information [file 41467_2017_1199_MOESM1_ESM.pdf]

Qie et al Supplementary Figure 1

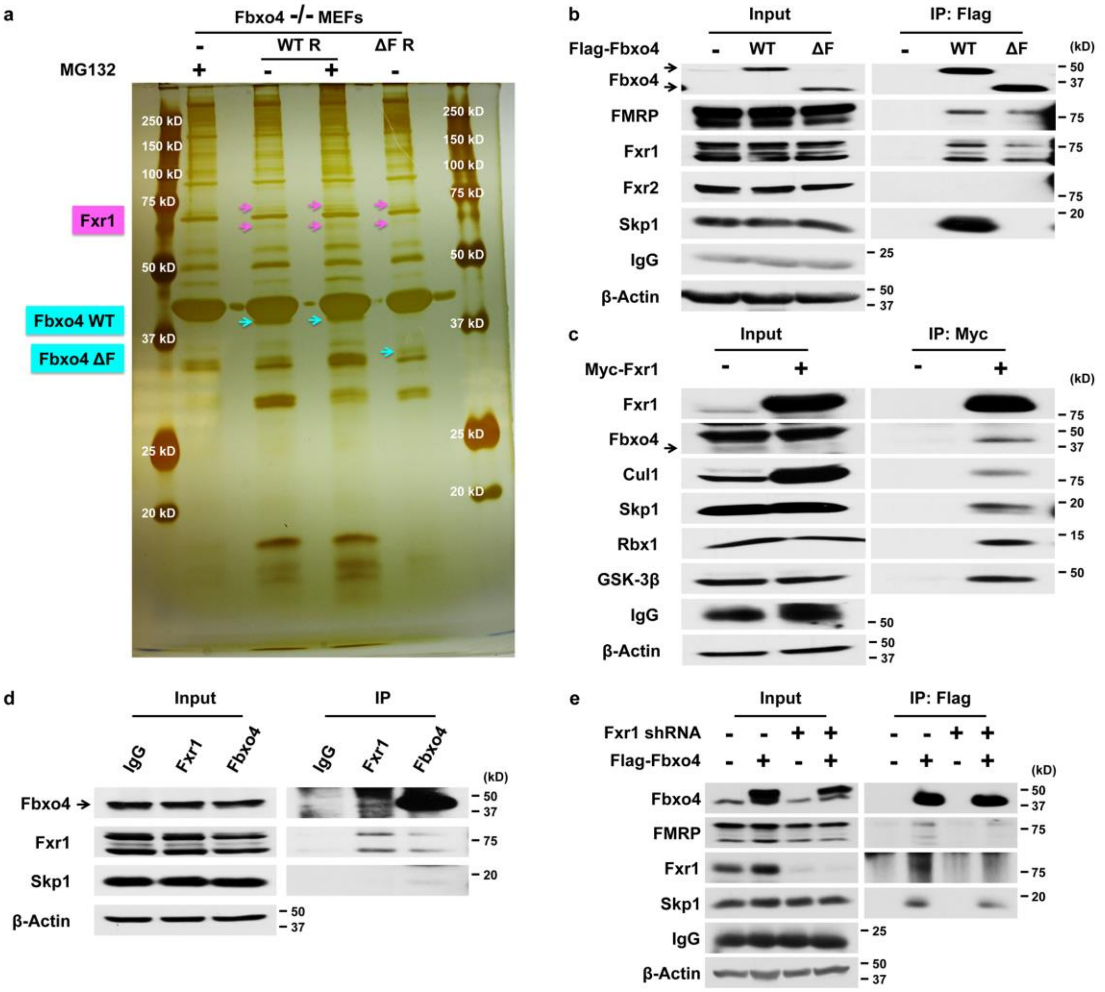

Supplementary Figure 1. Mass spectrometry analyses of Fbxo4 binding candidates and interaction between Fbxo4 and Fxr1.

(a) The silver stained gel before MS analyses. Magenta arrows show Fxr1 bands, turquoise arrows indicate the Fbxo4 WT and ΔF mutant. (b) Both WT and Fbxo4ΔF can pull-down endogenous Fxr1 and FMRP in HEK293T cells. (c) Fxr1 can pull-down the components of SCF complex in HEK293T cells. (d) Endogenous Fxr1 interacts with Fbxo4. (e) Fxr1 knockdown disrupts the interaction between FMRP and Fbxo4.

Qie et al Supplementary Figure 2

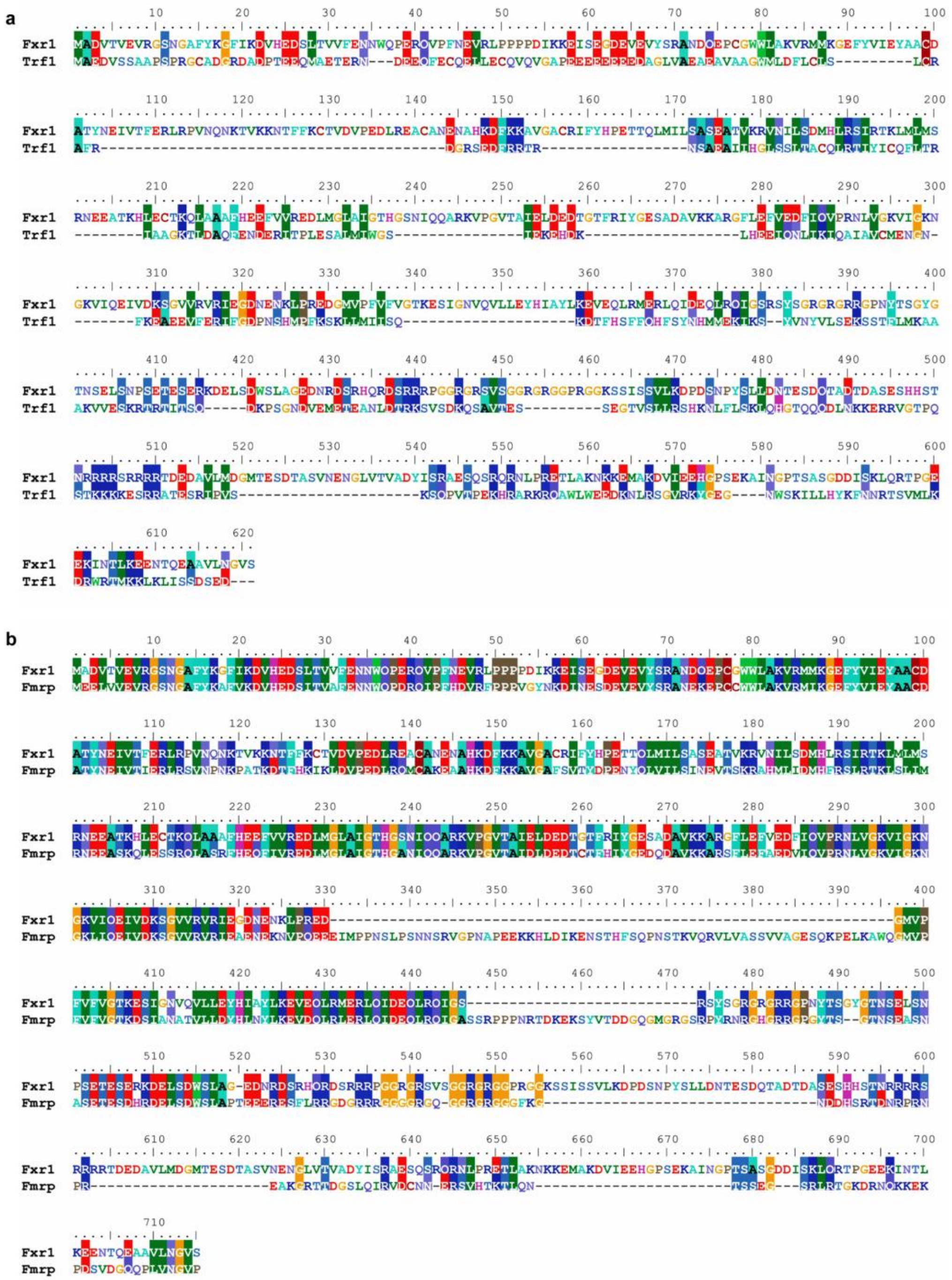

Supplementary Figure 2. BLOSUM64 matrix sequence analysis of Fxr1-Trf1 and Fxr1-FMRP.

(a) Sequence analysis of Fxr1-Trf1, the identities were 17% and similarities were 33%.

(b) Sequence analysis of Fxr1-FMRP, overall similarity between two proteins is 59%, but within the amino terminal domain used for the homology model, the identity is 81%.

Qie et al Supplementary Figure 3

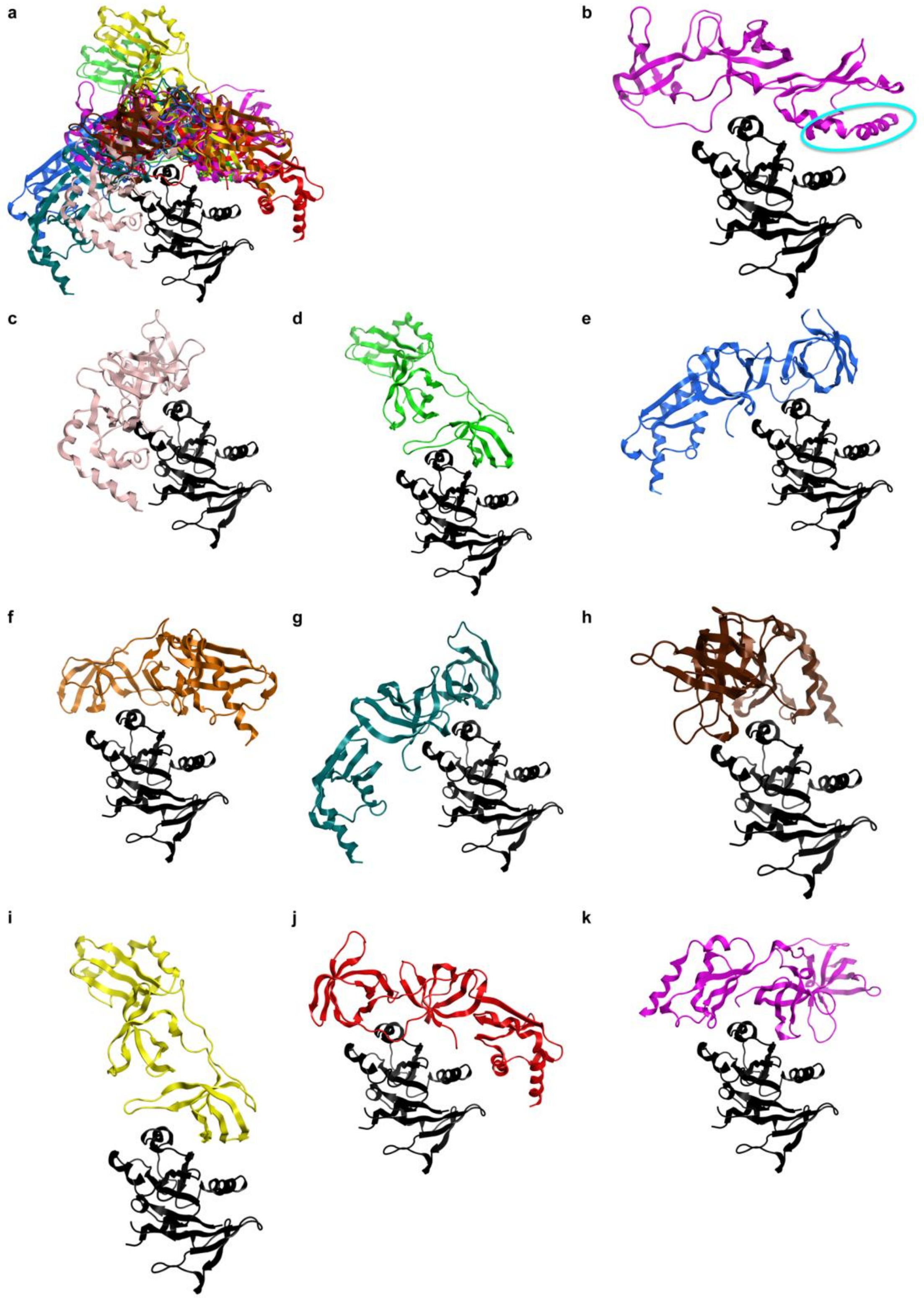

Supplementary Figure 3. Prediction of the binding interface between Fbxo4 and Fxr1 using bimolecular docking.

The Fbxo4 X-ray and Fxr1 homology model were analysed for the best possible dimer complex using ClusPro. (a) Superposition of the top 10 models. (b-k) Representative depiction of each model as individual dimer and in order from highest to lowest cluster size and weighted prediction score. In all images, Fbxo4 is depicted in black in the same orientation with Fxr1 in alternating colour for each model. Models 1 (b), 5 (f), and 9 (j) are highly similar in all dimensions, while models 1 (b), 4 (e), 5 (f), 7 (h), 9 (j) and 10 (k) use the same interface and only rotate about one axis. Model 1 (b) has the closest proximity of Fxr1 178-192 to Fbxo4 and this domain is circulated in turquoise oval. Model 2 (c), 4 (e), 6 (g), and 9 (j) indicate the pose driven by interactions with the termini of Fxr1 and are potential artifacts of molecular docking process.

Qie et al Supplementary Figure 4

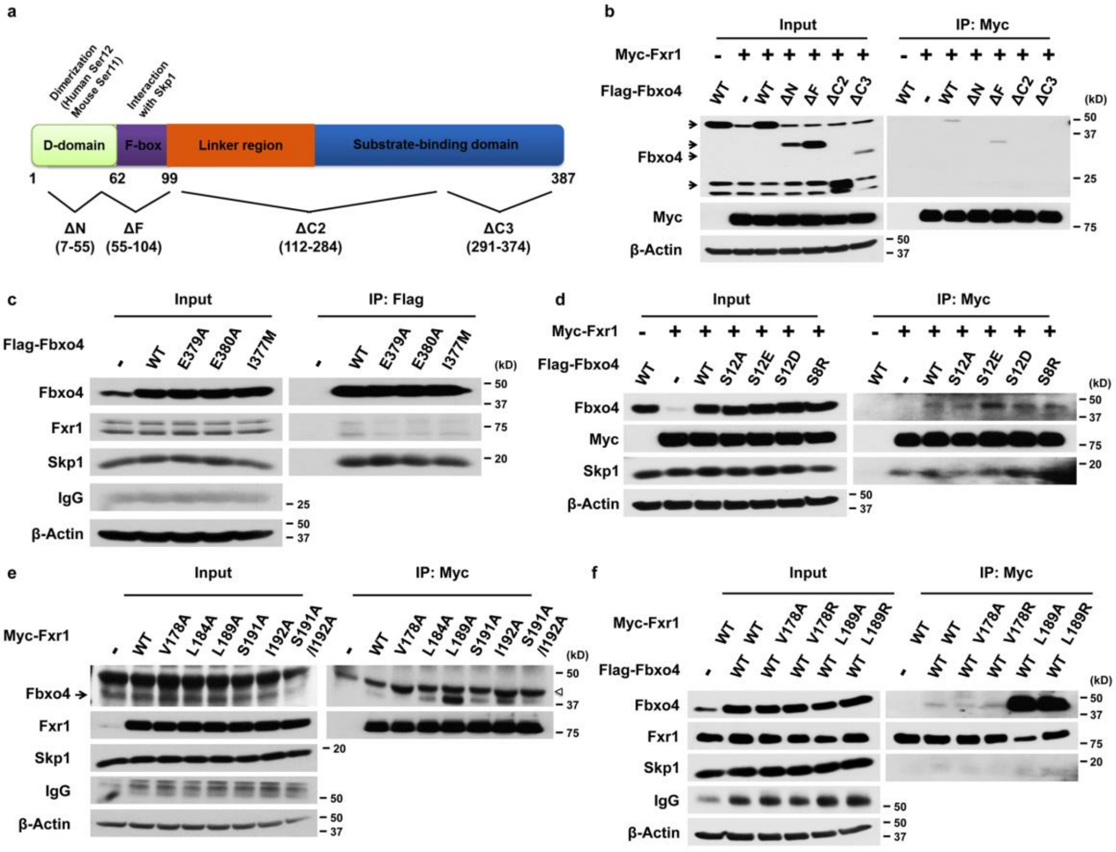

Supplementary Figure 4. Screening of mutations that affect the interaction between Fbxo4 and Fxr1.

(a) Schematic illustration of Fbxo4 structure and the deleting mutants of Fbxo4. (b) Fxr1 interacts with both WT and ΔF Fbxo4. (c) Fbxo4 E379A, E380A and I377M mutations disrupt the interaction between Fbxo4 and endogenous Fxr1. (d) Interaction between Fbxo4 mutants and Fxr1. (e) Fxr1 V178A suppresses while L189A Fxr1 enhances the interaction between Fxr1 and endogenous Fbxo4, empty triangle indicates nonspecific band. (f) Fxr1 mutants that disrupt or promote their interaction with Fbxo4.

Qie et al Supplementary Figure 5

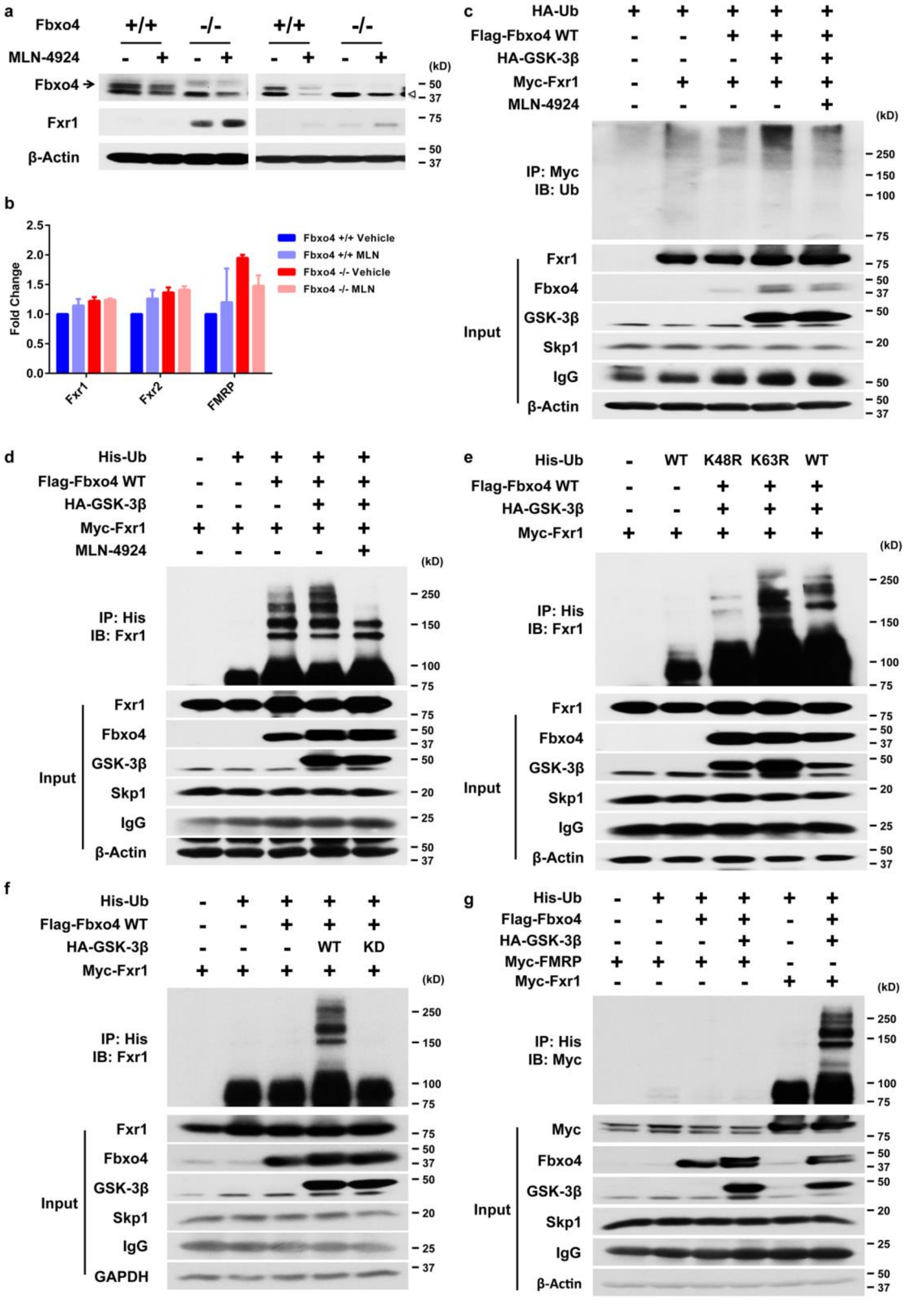

Supplementary Figure 5. Fbxo4 ubiquitylates Fxr1.

(a) Western blot indicates the expression of Fxr1 in two independent pairs of MEFs. Empty triangle indicates non-specific band from Fbxo4 antibody. (b) qRT-PCR analysis shows the expression of *Fxr1*, *Fxr2* and *FMRP* mRNAs in *Fbxo4*+/+ and -/- MEFs w/o MLN-4924 treatment (*n*=3). (c) *In vivo* ubiquitylation of Fxr1 by Fbxo4 (same membrane with that in Fig. 2c; stripped and re-developed). (d) His-tagged ubiquitin was used to permit denaturing immunoprecipitation of ubiquitylated Fxr1 proteins. (e) Fxr1 undergoes K48-linked ubiquitylation by Fbxo4 *in vivo*. (f) WT but not KD GSK3β promotes the ubiquitylation of Fxr1. (g) *In vivo* ubiquitylation assay of FMRP and Fxr1 by Fbxo4.

Qie et al Supplementary Figure 6

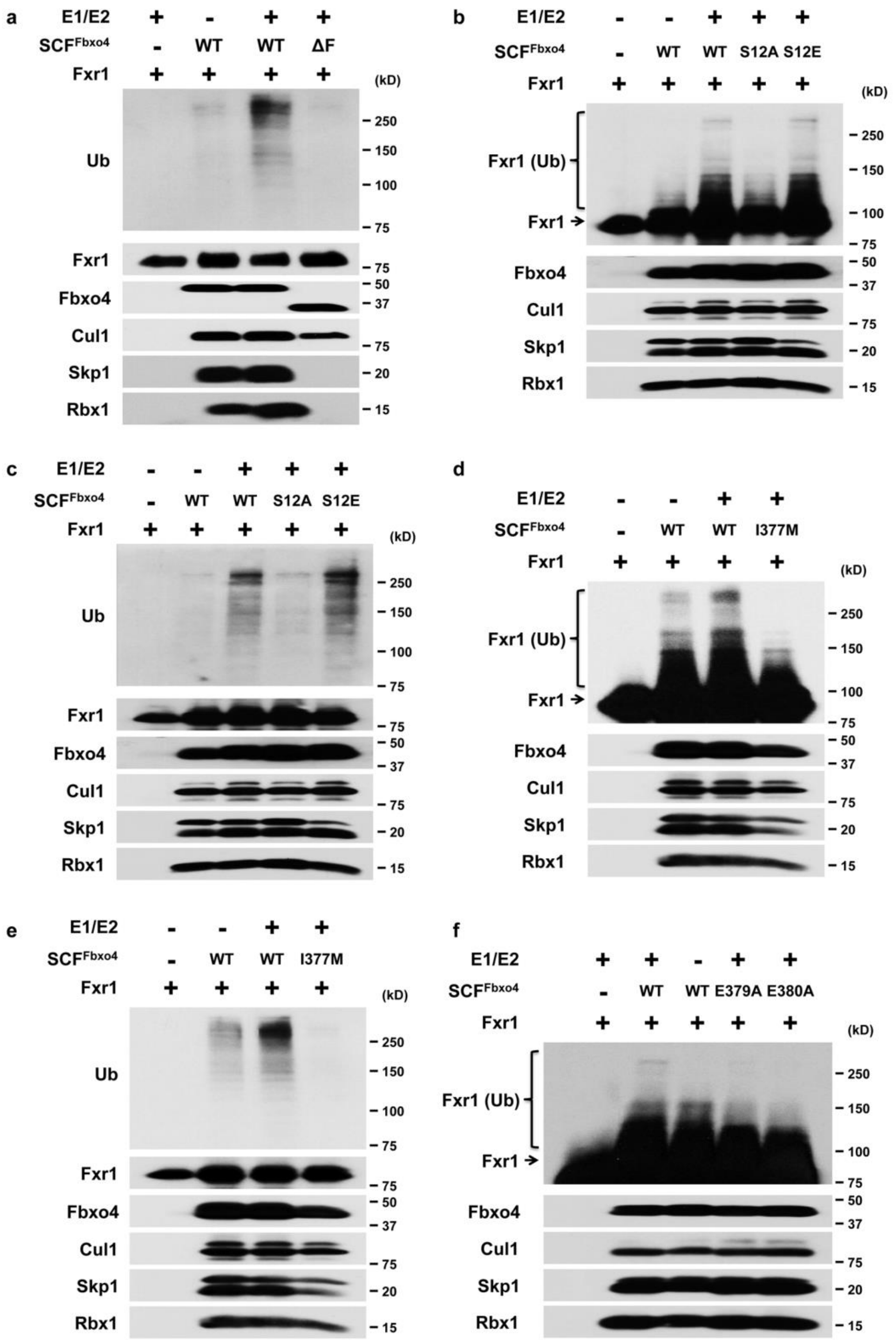

Supplementary Figure 6. Ubiquitylation of Fxr1 by both WT and mutant Fbxo4 *in vitro*.

(a) *In vitro* ubiquitylation of Fxr1 by Fbxo4 (same membrane with that in Fig. 2e; stripped and re-developed). (b & c) *In vitro* assay illustrates Fxr1 is ubiquitylated by both WT and S12E, but not by S12A Fbxo4. For ubiquitin blot in Panel (c), same membrane with Supplementary Fig. 6b, membrane was stripped and re-developed. (d & e) *In vitro* assay illustrates Fxr1 is ubiquitylated by WT, but not by I377M Fbxo4. For ubiquitin blot in Panel (e), same membrane with Supplementary Fig. 6d, membrane was stripped and re-developed. (f) *In vitro* assay illustrates Fxr1 is ubiquitylated by WT, but not by E379A and E380A Fbxo4.

Qie et al Supplementary Figure 7

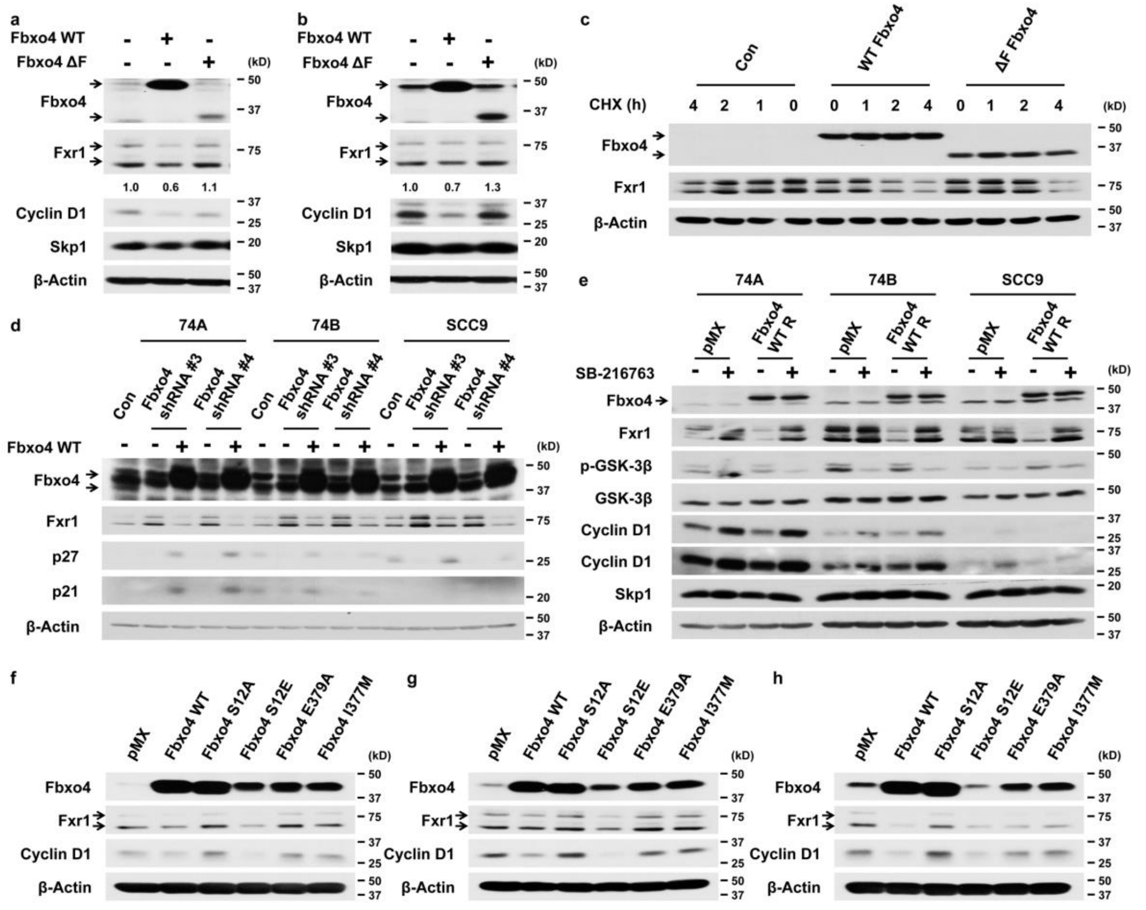

Supplementary Figure 7. Fbxo4 promotes the degradation of Fxr1 in a GSK3β-dependent manner.

(a & b) WT but not ΔF Fbxo4 suppresses Fxr1 expression in 74A (a) and 74B (b) cells. The numbers below Fxr1 bands indicate the band quantification. (c) Half-life of Fxr1 in HEK293T cells with WT and ΔF Fbxo4. (d) WT Fbxo4 antagonizes Fxr1 upregulation-mediated by Fbxo4 knockdown in HNSCC cells. (e) GSK3β inhibitor, SB-216763,

rescues Fbxo4-mediated Fxr1 degradation in HNSCC cells. (f-h) Overexpression of WT and S12E Fbxo4 suppresses Fxr1 expression in 74A (f), 74B (g) and SCC9 (h) cells.

Qie et al Supplementary Figure 8

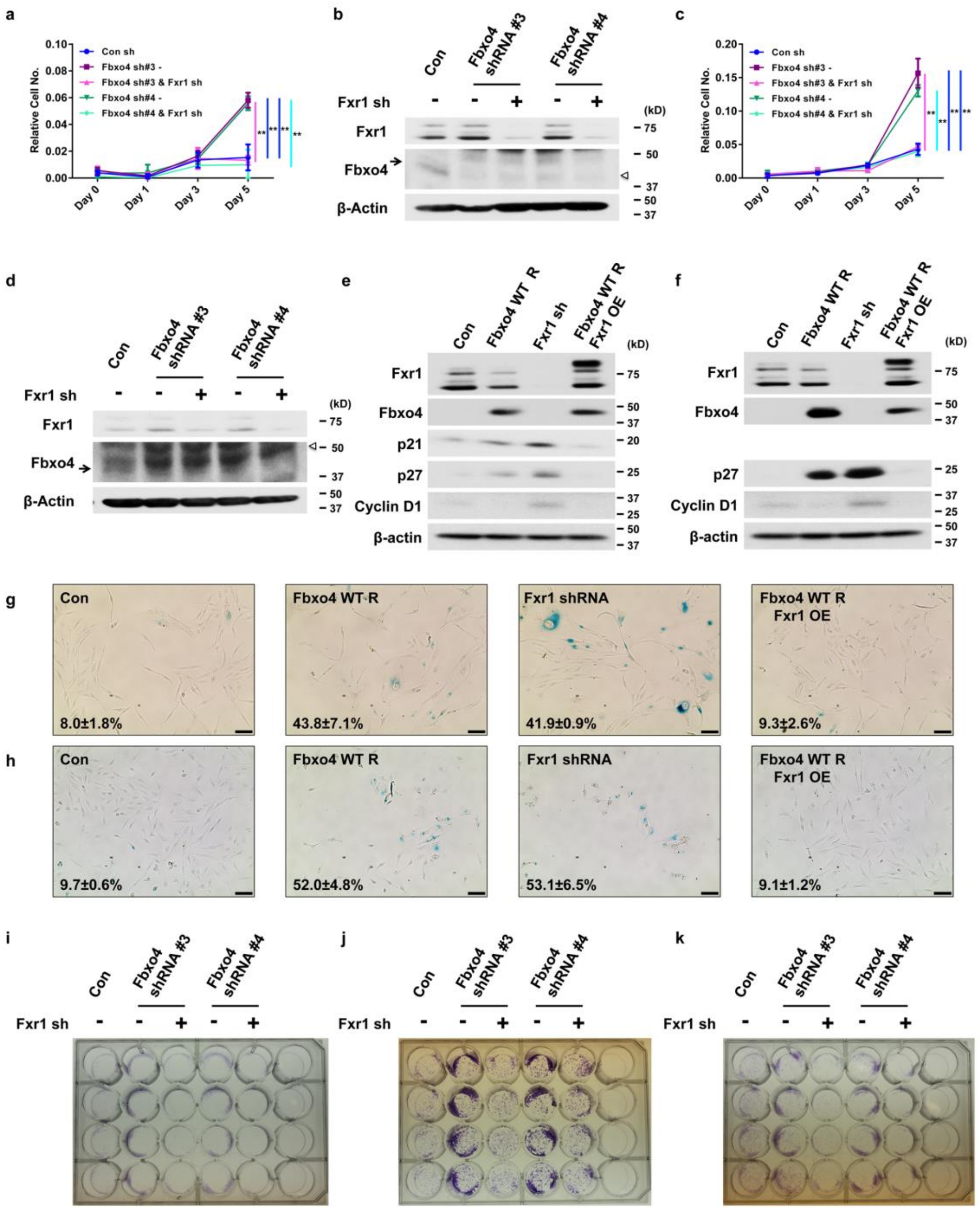

Supplementary Figure 8. Fxr1 promotes cell proliferation and inhibits senescence-induced by Fbxo4.

(a & c) *Fxr1* knockdown does reverse *Fbxo4* knockdown-induced cell proliferation. Growth curve of 74A (a) and SCC9 (c) cells. (b & d) Western blot shows *Fbxo4* and/or *Fxr1* knockdown in 74A (b) and SCC9 (d) cells. Empty triangle indicates nonspecific band. (e & f) Expression of p21 and p27 in 74A (e) and SCC9 (f) cells upon *Fbxo4* overexpression, *Fxr1* knockdown and both *Fbxo4* and *Fxr1* overexpression. (g & h) β-Gal staining indicates senescent cells in 74A (g) and SCC9 (h) cells upon *Fbxo4* overexpression, *Fxr1* knockdown and both *Fbxo4* and *Fxr1* overexpression. The numbers show the percentage of β-Gal positive cells in three independent experiments. (i-k) Plates show crystal violet staining on Day 5 before proliferation assay was performed in 74A (i), 74B (j) and SCC9 (k) cells. All data represent mean±s.d. and were analysed by Two-way ANOVA, followed by Fisher's LSD as Post Hoc Test (n=3). **, p<0.01. Scale bar, 10 μM.

Qie et al Supplementary Figure 9

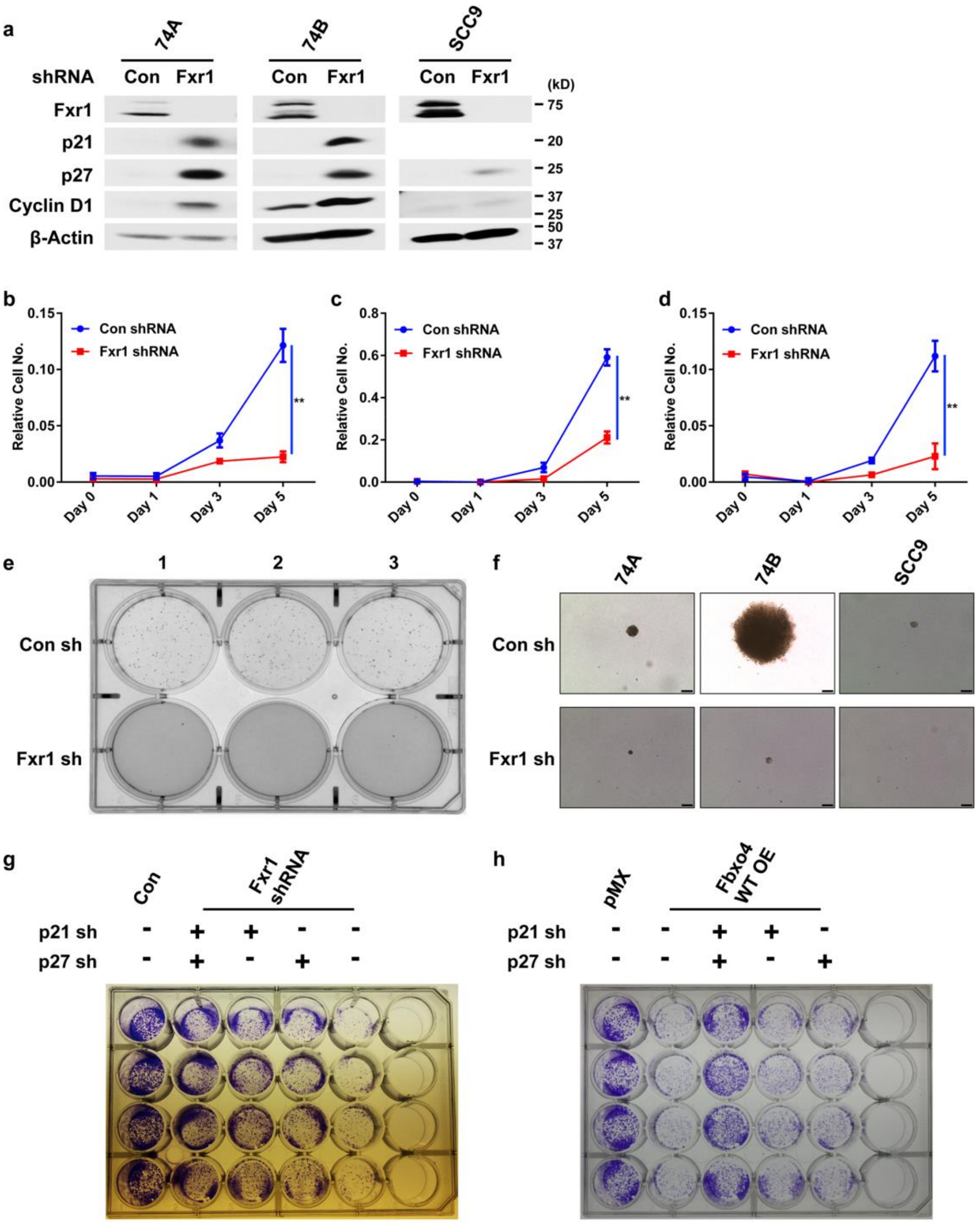

Supplementary Figure 9. *Fxr1* knockdown suppresses HNSCC cell proliferation and colony formation on soft agar.

(a) Western blots following *Fxr1* knockdown. (b-d) *Fxr1* knockdown reduces cell proliferation in 74A (b), 74B (c) and SCC9 (d) cells. (e & f) *Fxr1* knockdown suppresses colony formation in 74A, 74B and SCC9 cells. Panel (f) shows microscopic pictures. (g & h) Plates show crystal violet staining on Day 5 before proliferation assay was performed in *Fxr1* knockdown (g) and *Fbxo4* overexpressing (h) cells. All data represent mean±s.d. and were analysed by Two-way ANOVA (n=3). **, p<0.01. Scale bar, 10 μM.

Qie et al Supplementary Figure 10

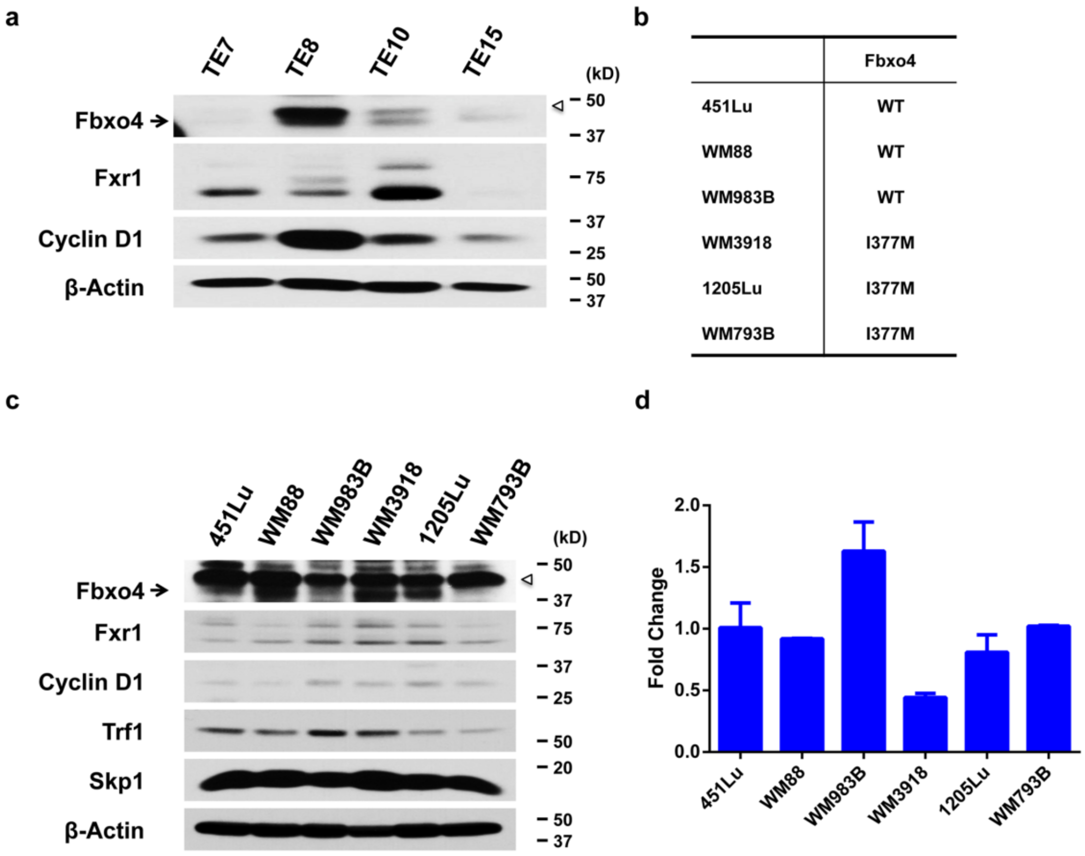

Supplementary Figure 10. Fxr1 is overexpressed in other human cancers.

(a) Fxr1 expression in oesophageal squamous carcinoma cells. Empty triangle indicates non-specific band from Fbxo4 antibody. (b) *Fbxo4* gene status in melanoma cells listed in Panel c. (c) Fxr1 protein levels in human melanoma cells. Empty triangle indicates non-specific band from Fbxo4 antibody. (d) *Fxr1* mRNA levels in human melanoma cells (n=3).

Qie et al Supplementary Figure 11

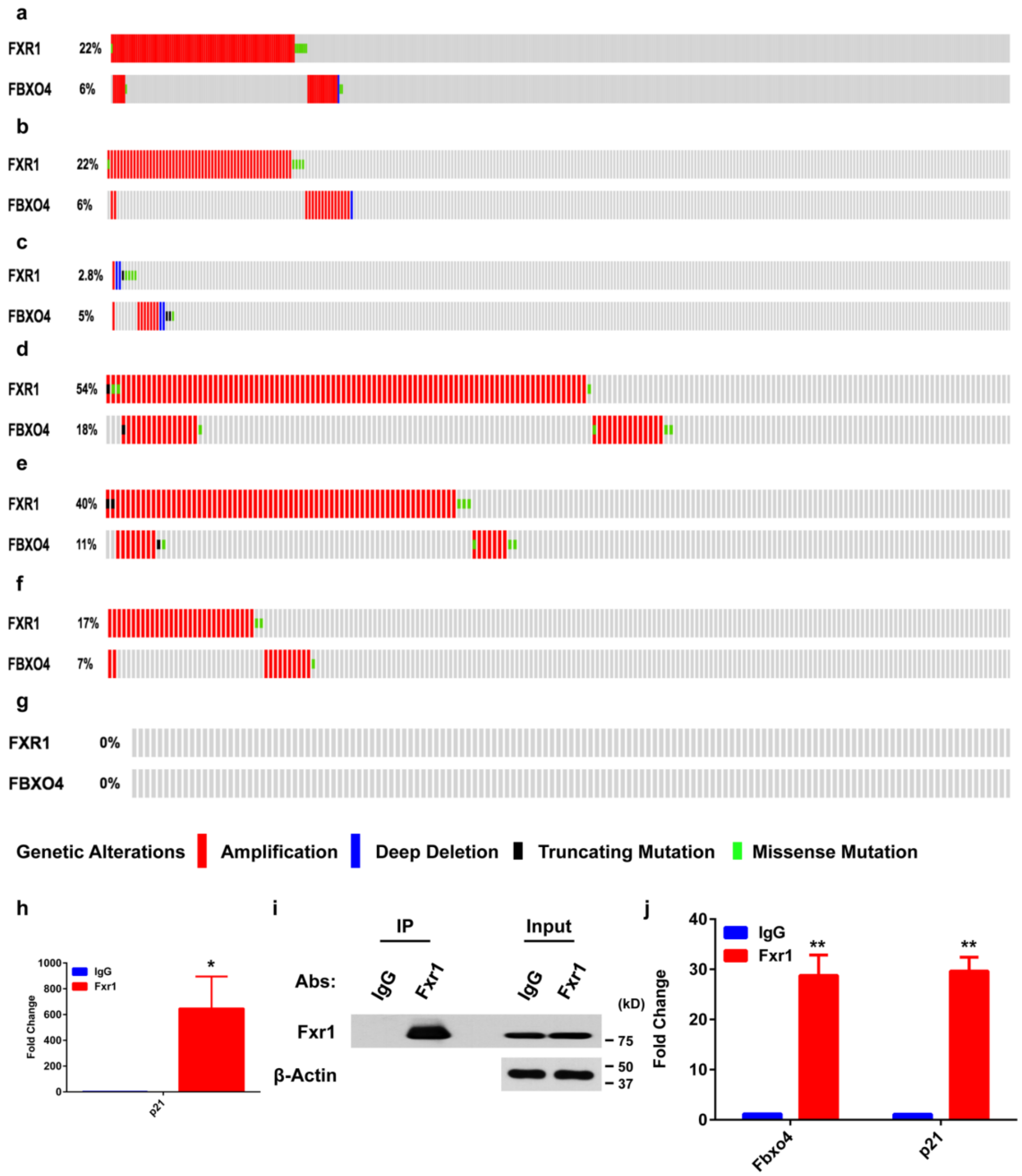

Supplementary Figure 11. Representative cBioPortal TCGA data analysis and RIP analysis.

(a) HNSCC: TCGA, Provisional, 504 patients/504 samples. (b) HNSCC: TCGA, Nature 2015, 279 patients/279 samples. (c) Skin Melanoma: TCGA, Provisional, 287 patients/287 samples. (d) Lung SCC: TCGA, Provisional, 177 patients/177 samples. (e) Lung SCC: TCGA, Nature 2012, 178 patients/178 samples. (f) Cervical SCC and Endocervical Adenocarcinoma: TCGA, Provisional, 191 patients/191 samples. (g) ESCC: UCLA, Nat Genet 2014, 137 patients/137 samples. (h) RIP analysis indicates Fxr1 can interact with *p21* mRNA in 74B cells. (i) Western blot of Fxr1 immunoprecipitation for RIP analysis in NIH3T3 cells with *Fxr1* overexpression. (j) RIP analysis shows the binding of *Fbxo4* mRNA by Fxr1 in NIH3T3 cells with ectopic *Fxr1* expression, *p21* was used as a positive control. All data represent mean±s.d. and were analysed by Student's *t* test (n=3). *, p<0.05; **, p<0.01.

Qie et al Supplementary Figure 12

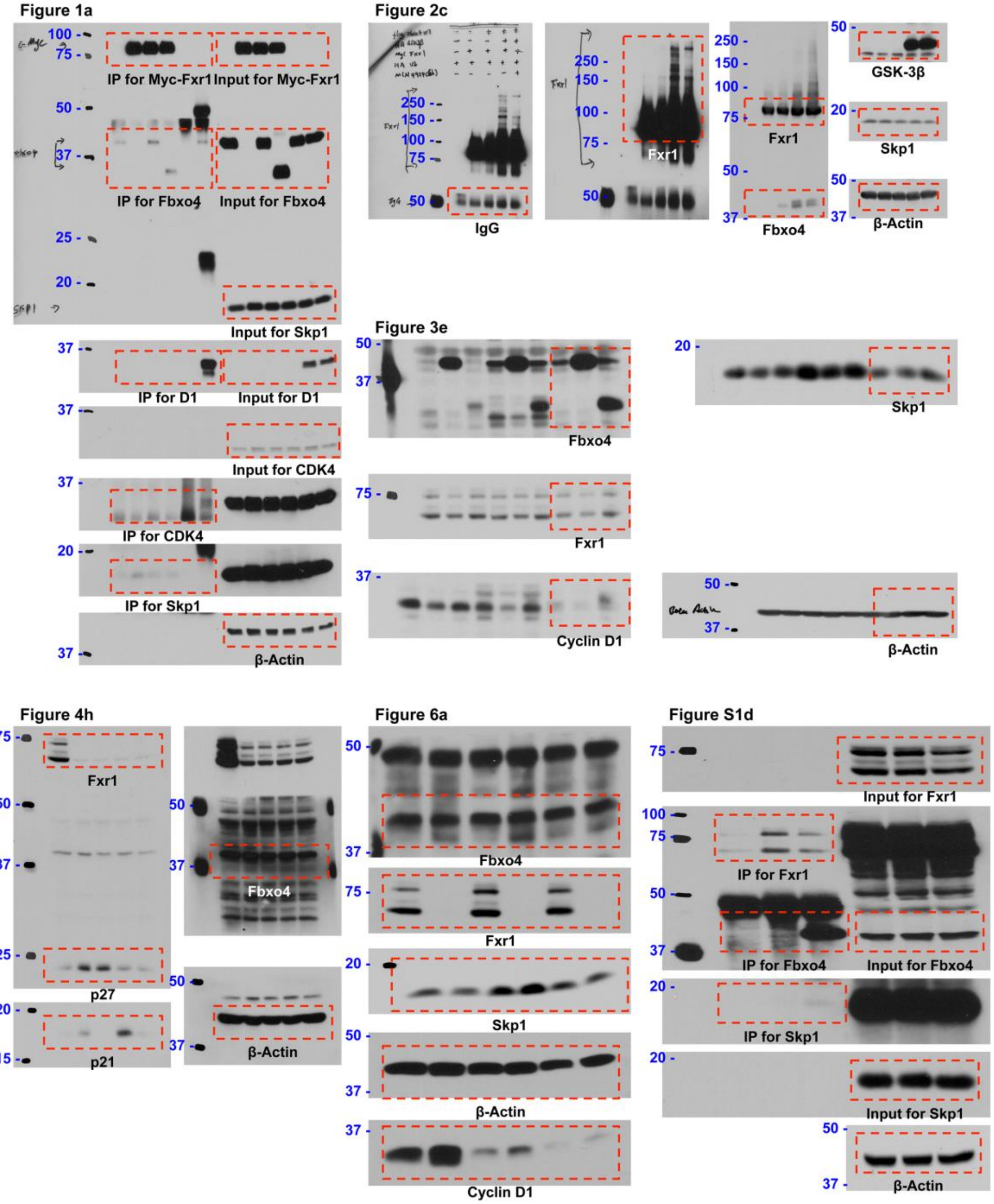

Supplementary Figure 12. Uncropped scans of the most important blots.

Supplementary Methods

Antibodies and chemicals

Antibody List:

Please refer to Supplementary Table 1

Chemical List:

MG-132 (20 μM in DMSO, Sigma-Aldrich, C2211)

MLN-4924 (10 μM in DMSO, Active Biochem, A-1139)

CHX (40 μg/ml in Ethanol, Sigma-Aldrich, C7698)

SB-216763 (25 μM in DMSO, Selleck Chemicals, S1075)

Plasmids

Fbxo4 plasmids were pcDNA3 vector with c-terminal flag-tagged WT, ΔN, ΔF, ΔC2, ΔC3, E379A, E380A, I377M, A341W/A354R, S12A, S12E, S12D, and S8R. As for requirement, flag-tagged Fbxo4 was transferred to the proposed vectors for further analyses. For virus production, *Fbxo4* WT, ΔF, S12A, S12E, E379A and I377M were cloned into pMX-puro vector (kindly provided by Dr. Xianxin Hua). pLenti-c-terminal myc-DDK-tagged *Fxr1* was genetically manipulated for overexpression, for example, Myc-Fxr1 was cloned into pcDNA3 vector for co-immunoprecipitation analyses. Cyclin D1, CDK4, HA-tagged GSK3β, HA-tagged Ubiquitin, and His-tagged Ubiquitin WT, K48R and K63R (kindly provided by Dr. Hui-kuan Lin) were used following the transfection protocol below. For site mutagenesis of Fxr1 (V178A, V178R, L184A, L189A, L189R, S191A, I192A and S191A/I192A), QuikChange II XL Site-Directed Mutagenesis Kit (Agilent Technologies) was utilized following manufacture's protocol.

Fbxo4 shRNAs (Clone ID: TRCN0000034321 and TRCN0000034322) and *p27* shRNA (Clone ID: TRCN0000039928) were bought from GE Dharmacon. *Fxr1* shRNA (Clone ID: TRCN0000159153) and *p21* shRNA (Clone ID: TRCN0000287021) were obtained from Sigma Mission.

Sf9 plasmids: Fbxo4 WT & ΔF, Cul1, Skp1, Rbx1, α-B-Crystallin, cyclin D1 and CDK4 were made by cloning the interested genes into either pVL1392 or pVL1393 vectors. Fxr1, Fbxo4 S12A, S12E, E379A and E380A were made by cloning the interested genes into pFastBac™ Vectors (Thermo Fisher Scientific Inc.), which can be used for baculovirus production in Sf9 cells.

Transfection and infection

For transfection: the transient expression of genes was achieved using lipofectamine with plus reagents according to the instructions from the manufacturer. In addition, PolyJet™ *in vitro* DNA transfection reagent was also used for DNA delivery.

Virus production for mammalian cells in HEK293T cells: to overexpress proteins in mouse cells, pMX vectors with interested genes were packaged in ψ2 retroviral vector; while for human cells, pMX vectors with interested genes were packaged in Qψ retroviral vector. For lentiviral packaging, *Fxr1* overexpression, *Fxr1*, *Fbxo4*, *p21* and *p27* knockdown, lentiviral backbone vectors were co-transfected with pMDLg/pRRE, CMV-**VSVG**, and RSV-**Rev** vectors. Generally, supernatant was collected 48 and 72 hours post-transfection. Infection was performed using 8 μg/ml polybrene if necessary, thereafter, cells were exposed to relative treatment.

Virus production in Sf9 cells: transfection was performed using the recombinant Bacimid with interested genes. Three to five days post-transfection, supernatant was collected for virus amplification (last for another 7 days). High titer viruses were utilized for Sf9 cell infection and protein purification.

MS analyses

Fbxo4 -/- MEFs w/o WT and ΔF Fbxo4 were exposed to medium containing 20 μM MG-132 for 6 hours. Cell pellets were lysed in Tween 20 lysis buffer (50 mM HEPES pH8.0, 150 mM NaCl, 2.5 mM EGTA, 1 mM EDTA, and 0.1% Tween 20 with protease and phosphatase inhibitors). After centrifugation, the supernatant was applied for immunoprecipitation with anti-FLAG® M2 affinity gel (A2220, Sigma-Aldrich) overnight. The following day, beads were washed and pulled-down proteins were eluted out using Flag peptides (F3290, Sigma-Aldrich). Eluted proteins were separated by SDS-PAGE gel and visualized with Pierce™ Silver Stain for Mass Spectrometry (24600, Thermo Fisher Scientific Inc). Excised bands were distained and sent out for LC-MS/MS in Taplin Biological Mass Spectrometry Facility at the Harvard Medical School. The data were analysed and proteins with at least two unique peptides were retained for further analysis.

Oncomine and cBioPortal analysis

For Oncomine analysis, *Fxr1* mRNA levels were searched in Oncomine™ database (https://www.oncomine.org/) with comparison of non-neoplastic versus tumour tissues.

For cBioPortal data mining, both *Fbxo4* and *Fxr1* genes were searched at the same time for the comparison of sequencing results as well as Copy Number Aberrations (CNAs) on the website: http://www.cbioportal.org/.

qRT-PCR

Upon treatment, total RNAs were extracted using RNeasy Mini Kit (Qiagen). One to two µg of total RNAs were reverse transcribed using SuperScript III Reverse Transcriptase according to the manufacturer's manual (Invitrogen). The cDNAs were diluted and run for quantitative analysis using predesigned and validated TaqMan probes with protocol indicated by Applied Biosystems. β-actin was used to normalize the RNA loading. Relative quantification was calculated using CFX Manager Software (Bio-Rad Laboratories, Inc.). The TaqMan probes are listed below: human *Fbxo4*: Hs00254777_m1, human *Fxr1*: Hs03845656_s1, human *p21*: Hs00355782_m1, human *TERC*: Hs03454202_s1, human *β-actin*: Hs01060665_g1, mouse *Fbxo4*: Mm01309961_m1, mouse *Fxr1*: Mm00484523_m1, mouse *p21*: Mm00432448_m1, mouse *TERC*: Mm01261365_s1, mouse *FMRP*: Mm01339582_m1, mouse *Fxr2*: Mm00839957_m1, and mouse *β-actin*: Mm00607939_S1.

CHX chase assay and quantification

Con shRNA and *Fbxo4* shRNA infected 74B cells were treated with 40 µg/ml CHX for indicated time. Whole cell lysate was collected for Western blot analysis. Band quantification was performed using Quantity One (Bio-Rad Laboratories, Inc.). Signals were normalized to β-actin.

***In vivo* and *in vitro* ubiquitylation assay**

For *in vivo* ubiquitylation assay, HEK293T cells were transiently transfected with myc-Fxr1 as well as WT or mutant Fbxo4 in the presence of HA-Ub or His-Ub w/o GSK3β. Twenty-four hours post-transfection, cells were collected, and boiled in 1% SDS at 95 °C for 10 min. Then, the reaction is quenched by 10% Triton X-100, finally, lysed in buffer (50 mM Tris pH7.5, 250 mM NaCl, 0.1% Triton X-100, 1 mM EDTA and 1 mM DTT) with protease and phosphatase inhibitors plus 5 mM NEM, and 20 μM MG132. After denaturing lysing, supernatant was incubated with anti-Myc agarose affinity gel or anti-His affinity resin. Thereafter, beads were washed and boiled; and SDS-PAGE gel was utilized to resolve the ubiquitylated proteins.

Sf9 cells were infected with vectors encoding Flag-Fbxo4 (either WT or mutants), α-B-crystallin, Cul1, Skp1, Rbx1 and Fxr1. The SCF complexes and Fxr1 protein were purified using anti-FLAG® M2 affinity gel or anti-Myc agarose affinity gel, respectively. Purified proteins were added in Eppendorf tube w/o E1, E2 (UbcH5a), ATP, and ubiquitin for 60 min at 37°C. After reaction, proteins were resolved on 10% SDS-PAGE gel and detected by relative antibodies.

Proliferation assay and soft agar assay

HNSCC cells with Con and *Fbxo4* shRNAs w/o *Fxr1* knockdown were seeded in 24-well plate at the number of $2x10^3$. Six, 24, 72 and 120 hours after seeding, cells were washed, then fixed with 4% paraformaldehyde in PBS for 15 min. Cells were washed and stained with 0.1% crystal violet for 20 min. Thereafter, staining solution was sucked out

and three times wash was performed using water. After final wash, the plates were air-dried. Extraction was done using 10% acetic acid with shaking for 20 min. The absorbance was measured at 590 nM. Signals from cells with six-hour seeding were set up as Day 0.

Luciferase reporter assay

The 74B cells with *Fxr1* knockdown and HEK293T cells with *Fxr1* overexpression were used for luciferase assay. Different segments of human *Fbxo4* 3'-UTRs were systematically identified and cloned into a luciferase reporter vector system, pLightswitch-3'-UTR from Switchgear Genomics. Empty vector, or *GAPDH* (negative control) and *p21* (positive control) were utilized as controls. Cells were incubated at 37°C prior to analysis. Twenty-four hours post-transfection, cells were lysed in LightSwitch Luciferase Assay kit following manufacturer's protocol. Luminescence signal was analysed via $20/20^n$ Luminometer (Turner Biosystems). The data were normalized to *GAPDH* 3'-UTR and 3'-UTR empty vector using LightSwitch normalization protocol (http://switchgeargenomics.com/sites/default/files/pdf/LightSwitch_3UTRnorm.pdf).

Supplementary Table 1 The Antibody List

Antibodies	Source	Catalogue No.	Dilution
Total Fbxo4	YenZym Antibodies	YZ1779	1:1,000
c-Myc	Santa Cruz	SC-40	1:10,000
CDK4	Santa Cruz	SC-749	1:200
Skp1	BD™	610530	1:5,000
β-actin	Sigma-Aldrich	A5316	1:50,000
Fxr1 (Western)	EMD Millipore	05-1529	1:10,000
Fxr1 (IP Western)	Cell Signaling	12295	1:1,000
Cyclin D1	EMD Millipore	CC12	1:500
p21	Santa Cruz	SC-397	1:200
GSK3β	BD™	610201	1:5,000
GSK3β	Cell Signaling	12456	1:1,000
Cul1	Santa Cruz	SC-11384	1:1,000
Rbx1	Cell Signaling	4397	1:1,000
p27	BD™	610242	1:2,000
pSer11/12 Fbxo4	YenZym Antibodies	YZ153	1:200
GAPDH	Cell Signaling	2118	1:1,000
Fxr1 (IHC)	Abcam	ab129089	1:50 ~ 1:150
FMRP	Abcam	ab17722	1:1,000
Fxr2	Cell Signaling	7098	1:1,000
Fxr1 (endoIP)	Proteintech	131-1-AP	1 ug/1 mg for IP
Ub	EMD Millipore	04-263	1:1,000
Trf1	Novus Bio	NB110-68281	1:1,000
