# Supplementary material for: Fbxo4-mediated degradation of Fxr1 suppresses tumorigenesis in head and neck squamous cell carcinoma
Source: Nat Commun. 2017 Nov 16;8:1534. doi: 10.1038/s41467-017-01199-8 (PMC5688124; doi:10.1038/s41467-017-01199-8)
Supplement: Supplementary file 3 — Description of Additional Supplementary Files [file 41467_2017_1199_MOESM3_ESM.pdf]

Description of Additional Supplementary Files

File Name: Supplementary Data 1
Description: C1_243 ClusPro bimolecular docking of Fbxo4 with Fxr1.

File Name: Supplementary Data 2
Description: C2_90 ClusPro bimolecular docking of Fbxo4 with Fxr1.

File Name: Supplementary Data 3
Description: C3_86 ClusPro bimolecular docking of Fbxo4 with Fxr1.

File Name: Supplementary Data 4
Description: C4_60 ClusPro bimolecular docking of Fbxo4 with Fxr1.

File Name: Supplementary Data 5
Description: C5_49 ClusPro bimolecular docking of Fbxo4 with Fxr1.

File Name: Supplementary Data 6
Description: C6_48 ClusPro bimolecular docking of Fbxo4 with Fxr1.

File Name: Supplementary Data 7
Description: C7_42 ClusPro bimolecular docking of Fbxo4 with Fxr1.

File Name: Supplementary Data 8
Description: C8_40 ClusPro bimolecular docking of Fbxo4 with Fxr1.

File Name: Supplementary Data 9
Description: C9_34 ClusPro bimolecular docking of Fbxo4 with Fxr1.

File Name: Supplementary Data 10
Description: C10_29 ClusPro bimolecular docking of Fbxo4 with Fxr1.

File Name: Supplementary Data 11
Description: Predicted AREs in 3'-UTR of Human Fbxo4 mRNA.
